# Age-Related Decline in Cervical Proprioception and Its Correlation with Functional Mobility and Limits of Stability Assessed Using Computerized Posturography: A Cross-Sectional Study Comparing Older (65+ Years) and Younger Adults

**DOI:** 10.3390/healthcare11131924

**Published:** 2023-07-03

**Authors:** Ravi Shankar Reddy, Batool Abdulelah Alkhamis, Junaid Ahmed Kirmani, Shadab Uddin, Waseem Mumtaz Ahamed, Fuzail Ahmad, Irshad Ahmad, Abdullah Raizah

**Affiliations:** 1 Department of Medical Rehabilitation Sciences, College of Applied Medical Sciences, King Khalid Universiry, Abha 61421, Saudi Arabia; balkamees@kku.edu.sa (B.A.A.); iabdulhamed@kku.edu.sa (I.A.); 2 Department of Physical Therapy, Faculty of Applied Medical Sciences, Jazan University, Jazan 45142, Saudi Arabia; jkirmani@jazanu.edu.sa (J.A.K.); ssabauddin@jazanu.edu.sa (S.U.); wahamed@jazanu.edu.sa (W.M.A.); 3 Respiratory Care Department, College of Applied Sciences, Almaarefa University, Riyadh 13713, Saudi Arabia; fahmad@mcst.edu.sa; 4 Department of Orthopaedics, College of Medicine, King Khalid University, Abha 61421, Saudi Arabia; araizizah@kku.edu.sa

**Keywords:** older adults, proprioception, balance, limits of stability, joint position sense

## Abstract

Cervical proprioception and its implications on postural stability are crucial in older adults. Understanding their relationship is important in understanding and preventing falls in older adults. This research aims to evaluate the proprioceptive, functional mobility, and limits of stability (LOS) variables among two age groups: individuals aged 65 and above and those below 65. A secondary goal of the study is to analyze the relationship between cervical proprioception, functional mobility, and the LOS. Methods: In this cross-sectional study, 100 participants each were included in the older and younger groups. Researchers employed the target reposition technique to assess cervical proprioception and measured the joint position error (JPE) in degrees. Functional mobility was estimated using the Berg balance scale (BBS) and timed up-and-go test (TUG). In addition, dynamic posturography was utilized to evaluate variables related to the LOS, including reaction time, maximum excursion, and directional control. Results: The magnitudes of the mean cervical JPE are larger (*p* < 0.001), and functional mobility (*p* < 0.001) and the LOS (*p* < 0.001) are impaired in older individuals compared to the younger ones. The cervical proprioception is significantly associated with functional mobility (*p* < 0.001), and the LOS (*p* < 0.001). Conclusion: In older adults aged above 65 years, cervical proprioception, functional mobility, and the LOS are impaired. Older adults with greater cervical JPE had more impaired functional mobility and LOS parameters. When evaluating or treating older adults with problems with their balance or falls, these factors should be considered.

## 1. Introduction

Falls pose a widespread and considerable health threat to older individuals. Due to the natural aging process, the risk of falls increases, which can lead to a decreased quality of life [1]. The annual incidence of falls among those over 65 is between 24 and 40 percent [2]. Given the longer lifespans, it is more crucial to notice the differences among older persons and identify life changes in later years [3]. After a fall, one-third of older persons stop engaging in specific hobbies, have fewer social connections, and feel less confident about themselves [3]. For the older person, these exercise and social engagement limitations lead to a decline in quality of life, a loss of autonomy, and increased dependency on others [4]. Therefore, a fall incidence is a defining moment for many older persons [5]. If no preventive measures are implemented, fall-related injuries are expected to double by 2030 [5,6]. Effective proprioception and postural control are crucial for maintaining body equilibrium and preventing falls in older adults [7].

Cervical proprioception plays a crucial role in maintaining the stability and function of the neck and head [8]. Proprioceptors in the cervical spine provide vital sensory information to the brain, which helps to control posture, balance, and movement [9]. Proper cervical proprioception is essential for the accurate and coordinated movement of the head, neck, and upper extremities, and for maintaining body position and spatial orientation [10]. It is an important sensory mechanism that helps to maintain postural control and balance during various activities of daily living [11,12,13]. As individuals age, the ability of the neck muscles and joints to sense proprioceptive feedback may decline, leading to decreased postural control and an increased risk of falls [14]. The reduction of cervical proprioception could be attributed to various age-related transformations in the structure and functionality of the neck muscles and joints, and alterations in the central nervous system’s capacity to interpret sensory input [15].

Assessing postural control and balance can be carried out through the limits of stability (LOS) test, which evaluates an individual’s capacity to maintain equilibrium while shifting their weight in various directions [16]. As previous research has revealed, there is a well-established correlation between impaired postural control and a higher risk of falls [17,18,19]. In particular, Johansson and colleagues discovered that the amplitude of postural sway, evaluated in millimeters squared, could predict falls in older persons and reflect their functional mobility [20]. It is essential to have a comprehensive awareness of the systems that contribute to maintaining postural control to reduce the risk of falling and prevent falls from occurring [11]. Multiple authors have established a link between reduced proprioceptive ability and modified functional mobility [21,22].

Older individuals may have a reduced ability to maintain stability during the LOS test, which may be related to declines in cervical proprioception [23]. Improving cervical proprioception through targeted exercises and other interventions may help to enhance functional mobility and reduce the risk of falls in individuals with age-related changes or other impairments affecting their cervical proprioception [10]. Gaining insight into the significance of cervical proprioception and how it affects functional balance and the LOS in older adults can provide valuable information for designing interventions to enhance balance and minimize the likelihood of falls among this demographic. Therefore, the study’s primary objective is to estimate the proprioceptive, functional mobility, and LOS variables in the older group (above 65 years) and compare them with younger individuals (below 65 years of age). The secondary objective was to assess the association between cervical proprioception, functional mobility, and the LOS.

## 2. Materials and Methods

### 2.1. Design and Subjects

The study involved 200 participants, divided into two groups based on age: the older group, with 100 individuals over 65 years old, and the younger group, with 100 individuals under 65 years old. The participants were selected using a convenience sampling method from the university campus and the local community. The study only included healthy individuals aged 40–80 years and excluded those with a history of musculoskeletal injury, diabetes, stroke, dizziness, vestibular dysfunction, or surgical hardware.

Participants aged below 65 years were categorized as the ‘younger’ group compared to the older adults (above 65 years) in this study. While the term ‘young’ may be subjective, we selected individuals below 65 years to capture a broader range of adults who are within their working-age and active adult years. This age range was chosen to allow for meaningful comparisons and to explore the age-related differences in cervical proprioception, functional mobility, and limits of stability. Although alternative age ranges, such as 18–40 years, are commonly used to define young adults, we opted for a broader age range to ensure the inclusion of a representative sample of adults within the study’s objectives, available resources, and feasibility considerations.

The Declaration of Helsinki is a set of guidelines for conducting medical research that outlines ethical principles and standards for protecting human subjects. In this study, the researchers followed these guidelines, ensuring all participants signed a written consent form before participating. The study received approval from a King Khalid University ethical committee (REC# 16/10/156). By adhering to these ethical principles and obtaining approval from the ethical committee, the researchers ensured the participants’ safety and well-being, and maintained the study’s integrity.

### 2.2. Assessment of Cervical Joint Position Sense

**Figure 1:**
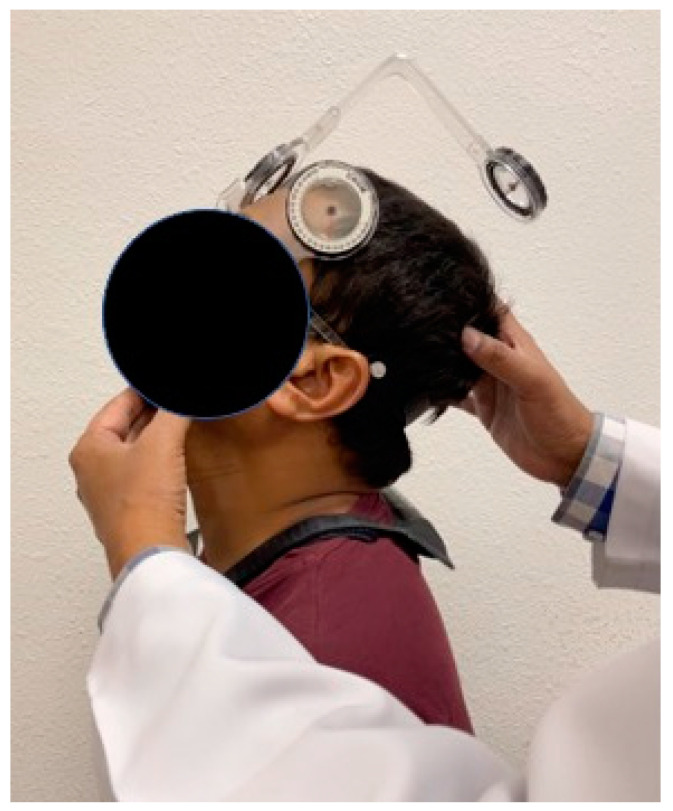
Cervical joint position sense evaluation using a CROM device.

The cervical range of motion (CROM) device is a widely used tool for evaluating cervical proprioception, which is the sense of position and movement of the neck. A prior study by Reddy et al. [24] demonstrated that the CROM device exhibited acceptable test–retest reliability in assessing cervical proprioception in individuals with neck pain and asymptomatic individuals (ICC values ranging from 0.66 to 0.93) [24]. The method of evaluation using the CROM device involves measuring the cervical range of motion in two planes of movement: flexion–extension and rotation, as shown in Figure 1.

The CROM device consists of a head-mounted device with an inclinometer that measures the angle of the head in relation to the trunk. The device is placed on the forehead of the individual being evaluated, and the individual is asked to perform a series of movements. In contrast, the device records the angle of the head in each plane of movement. Several studies have investigated the reliability and validity of the CROM device and found that it is a consistent and accurate method for measuring the cervical range of motion and proprioception [25]. This device has been tested on healthy individuals and individuals with neck pain, demonstrating its applicability in various clinical settings. With this device, clinicians can reliably and accurately measure the cervical range of motion and proprioception, which can help guide treatment decisions and monitor progress over time [25]. We adopted the target reposition sense technique for testing cervical JPS. The subjects were asked to sit on the chair with their feet flat on the ground. The target was selected as 25° in each plane of motion tested [26]. The individual being evaluated is asked to move their head and neck to a target position (25° of flexion or extension or rotation left or right), hold that position for five seconds, and then return their head to the starting position. Participants were instructed to actively use their neck muscles to move their head and neck to a specific target position. To evaluate the accuracy of cervical joint position error (JPE) or proprioception, the researchers measured the degree of difference between the intended target position and the actual position of the head and neck.

### 2.3. Assessment of Limits of Stability

**Figure 2:**
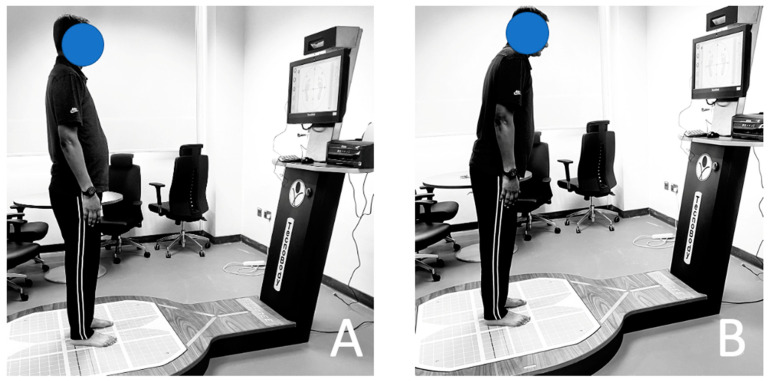
Limits of stability assessment: (**A**) starting and (**B**) moving to a target.

Dynamic posturography using an iso-free technology device is a modern tool for measuring the limits of stability (LOS), the maximum distance an individual can lean without losing balance. The LOS refer to the maximum distance or angles an individual can lean or shift their body’s center of gravity without losing balance or stepping to prevent a fall [26]. This device uses a force plate mounted on a movable platform to simulate dynamic movements, allowing for a more accurate assessment of the LOS. At the same time, this method has advantages over traditional posturography. The patient stood on the stabilometric force platform with both feet together while the posturography device displayed targets on the screen to guide them. This was carried out so that the device could determine the subject’s LOS. It was requested of the participant that they move their center of mass in the direction of the goal without shifting their feet in any of the eight distinct directions. The tool recorded the amount of sway required to achieve the goal, and a score was awarded for it. (Figure 2).

The LOS tests are a method used to evaluate an individual’s postural control and stability in response to different challenges. The study assessed the LOS by measuring three specific parameters: reaction time, maximum excursion, and directional control [16]. Reaction time refers to the time taken by an individual to respond to an unexpected disturbance or perturbation that affects their balance. This parameter indicates how quickly an individual can adjust their posture in response to external factors, such as sudden movement or uneven terrain [16]. The maximum excursion is the distance an individual can lean or shift their weight in different directions without losing balance or stability. This parameter provides information about the range of motion that an individual can tolerate while maintaining their balance, and is a crucial aspect of postural control [16]. Directional control, the third parameter of the LOS test, assesses an individual’s ability to maintain postural stability while shifting their weight in different directions. This parameter is particularly relevant in activities that require the individual to make rapid changes in direction or position, such as sports or activities of daily living [16].

Researchers can obtain a detailed assessment of an individual’s postural control and stability by measuring these three parameters. This information can be used to develop targeted interventions to improve postural stability and reduce the risk of falls and other injuries. The study findings can also contribute to developing effective rehabilitation programs for patients with cervical spine issues or other conditions that affect postural control.

### 2.4. Assessment of Functional Mobility

#### 2.4.1. Berg Balance Scale (BBS)

The Berg balance scale is a standardized assessment that involves 14 functional tasks, such as standing, transferring, and reaching, which are scored on a 5-point scale [27]. Research has shown that this test is reliable and valid for predicting falls in the elderly population, with scores below 45 indicating an increased fall risk and scores below 40 indicating a high fall risk [28]. Regular use of the Berg balance scale can identify individuals who may benefit from targeted interventions to improve balance and reduce the risk of falls in the elderly population. The reliability and validity of the Berg balance scale have been confirmed through several studies, with ICC values ranging from 0.98 to 0.99 [27]. Due to its effectiveness and simplicity, the Berg balance scale is a widely used clinical tool to assess balance and fall risk in the elderly population. Clinicians can use the test results to develop appropriate interventions, such as exercise programs or modifications to the individual’s environment, to improve balance and reduce the risk of falls [27]. Overall, the Berg balance scale is an important tool for promoting the safety and well-being of elderly individuals.

#### 2.4.2. Timed Up and Go (TUG) Test

The Timed Up and Go (TUG) test is commonly used to assess functional mobility in older adults [29]. The test involves having participants sit in a standard armchair, stand up, walk a distance of three meters, turn around, and sit back down in the chair [29]. The time taken to complete the task is recorded, and the test is repeated three times, with the average score used for analysis. The TUG test has high test–retest reliability, with an ICC value of 0.99 [29]. In older adults, completing the TUG test in less than 12 s is considered to be indicative of lower fall risk. Therefore, the TUG test is important for clinicians and rehabilitation therapists to assess functional mobility and fall risk in older adults [30].

### 2.5. Sample Size Calculation

To determine the appropriate sample size for our study, we estimated the sample size based on a conservative effect size derived from a systematic review [31]. The review reported a standardized mean difference (SMD) of 1.19 (95% confidence interval [CI] 0.71–2.63, *p* < 0.001) for joint position sense in patients with neck pain. We adopted the lower confidence interval limit (0.94) as the effect size (Cohen’s d) between the older and younger groups in our study. With a significance level of 5% (α = 0.05) and a power of 95% (β = 0.05), we calculated sample size using a two-sided t-test for two independent samples. Using G*Power Software version 3.1.9 for the calculation, we determined that a total sample size of 186 participants would be sufficient to detect a significant difference in cervical proprioception between the two age groups, considering the potential loss of participants during the study. Therefore, we included 200 participants (100 in each age group) in the analysis to ensure adequate statistical power and reliable findings.

### 2.6. Statistical Analysis

The Shapiro–Wilk tests were conducted to evaluate the normality of the data distribution in the study variables, and it was found that they followed a normal distribution. The study variables, including cervical proprioception and balance characteristics, were presented using mean and standard deviation measures. To compare differences in these variables between participants aged above and below 65 years, the researchers employed an independent *t*-test. The correlation between cervical proprioception, functional mobility, and LOS variables was analyzed with Pearson’s correlation coefficient (r). It was ranked low when the correlation was between 0.20 and 0.30, moderate between 0.31 and 0.69, and high between 0.70 and 1. Version 22.0 of SPSS was used to carry out the statistical analysis.

## 3. Results

Table 1 presents the physical and demographic characteristics of the study participants, with a total of 100 participants in each age group (older group (>65 years) and younger group (<65 years)).

The results show that the mean age of the older group was significantly higher than that of the younger group (69.53 ± 4.32 vs. 52.30 ± 4.23, *p* < 0.001), indicating a clear difference in age distribution between the two groups. In terms of gender, the older group had more males than females (59:41), while the younger group had a more equal gender distribution (52:48) (*p* < 0.001). Additionally, the study found that the mean height of the younger group was significantly greater than that of the older group (1.73 ± 0.05 vs. 1.68 ± 0.09, *p* = 0.002), while, in contrast, eight members of the older group were slightly taller than the younger group (71.24 ± 5.96 vs. 69.58 ± 5.24, *p* = 0.030). However, the mean BMI of the two groups did not differ significantly (24.50 ± 3.80 vs. 23.38 ± 2.74, *p* = 0.201).

Table 2 presents the descriptive characteristics of the cervical joint position sense, mobility, and LOS tests for the two age groups (the older group (>65 years) and the younger group (<65 years)). The results show significant differences between the two groups across all parameters measured.

In terms of cervical joint position sense, the older group had significantly higher JPE values in all directions (flexion, extension, right rotation, and left rotation) compared to the younger group (*p* < 0.001). This indicates that the older group had a reduced ability to accurately perceive the position of their head and neck.

The BBS and the TUG test were used to evaluate functional mobility, with the younger group performing significantly better than the older group (*p* < 0.001). Additionally, the older group had a longer TUG test duration, indicating slower functional mobility.

**Figure 3:**
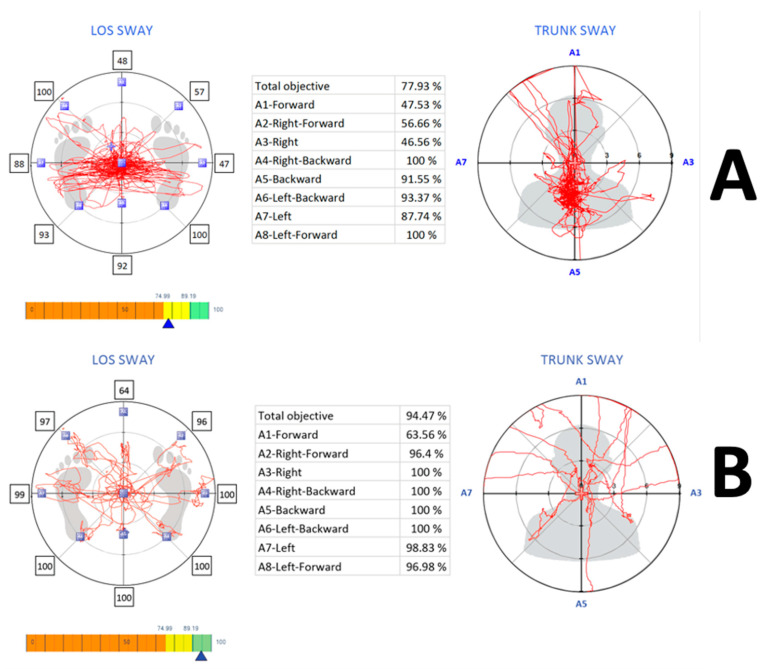
Limits of stability in (**A**) the older group (>65 years) and (**B**) the younger group (<65 years).

The LOS tests, which measured reaction time, maximum excursion, and directional control, also showed significant differences between the two groups (*p* < 0.001). The younger group had faster reaction times, greater maximum excursion, and better directional control than the older group, indicating better postural stability (Figure 3).

**Figure 4:**
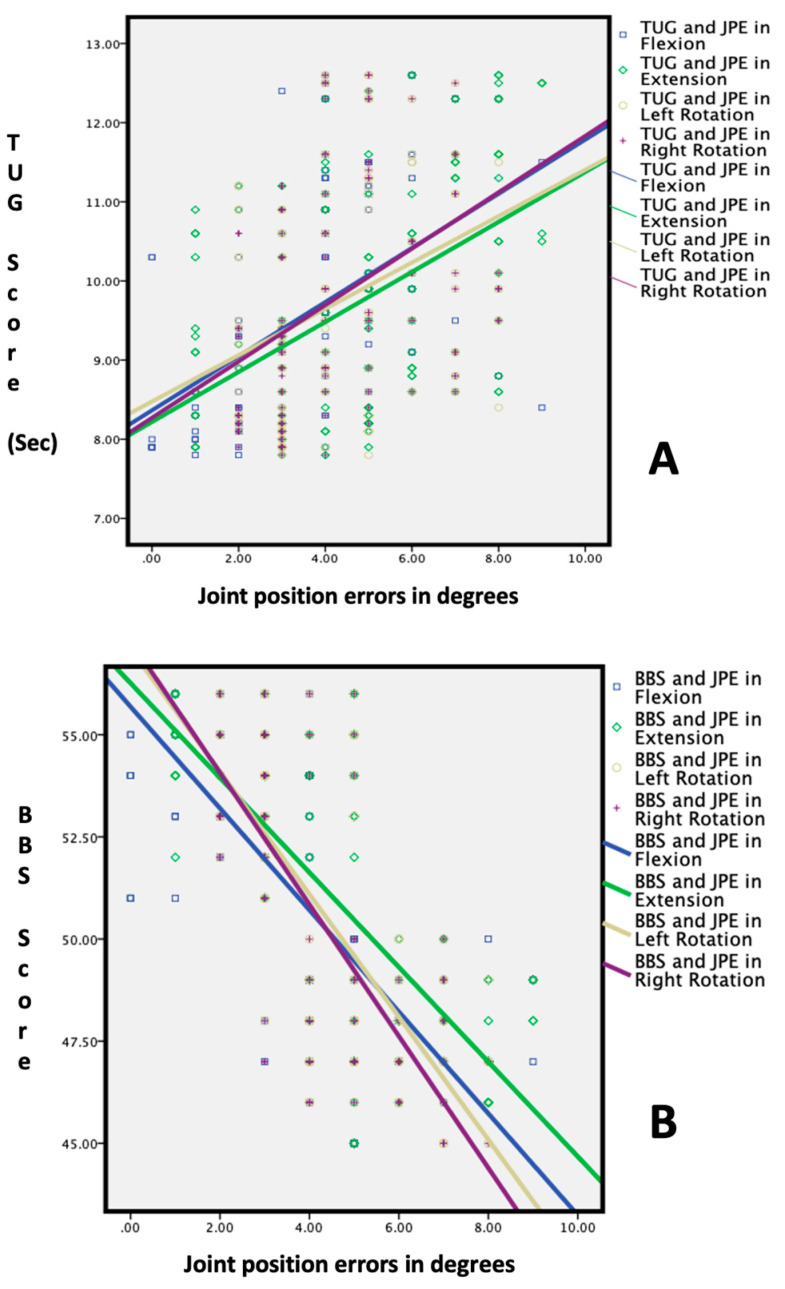
Correlation between cervical proprioception errors and functional balance tests: (**A**) Berg balance test score vs. joint position errors; and (**B**) Timed Up and Go test vs. joint position errors.

**Figure 5:**
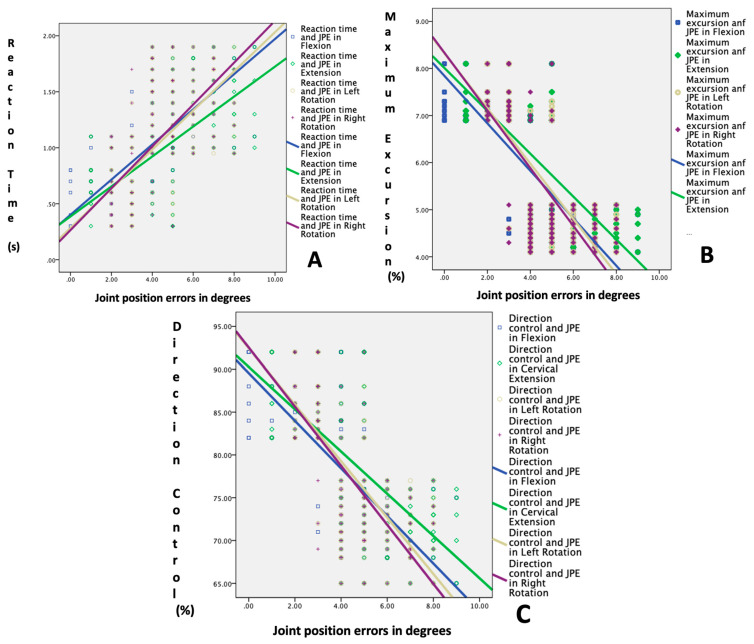
Correlation between cervical joint position errors and LOS tests: (**A**) reaction time (sec) vs. joint position errors; (**B**) maximum excursion % vs. joint position errors; and (**C**) direction control % vs. joint position errors.

Table 3 and Figure 4 and Figure 5 show the relationship between the cervical JPE and the balance and LOS tests in adults above and below 65. The results indicate significant negative correlations between the JPE in all directions (flexion, extension, right rotation, and left rotation) and the BBS test score, indicating that higher JPE values are associated with poorer balance performance in both age groups (*p* < 0.01).

The TUG test showed significant positive correlations with the JPE in all directions in both age groups (*p* < 0.01), indicating that higher JPE values are associated with a longer TUG test duration, indicating slower functional mobility. Furthermore, there were significant positive correlations between the JPE values and reaction time, indicating that higher JPE values are associated with longer reaction times in both age groups (*p* < 0.01). Additionally, there were significant negative correlations between the JPE values and maximum excursion and directional control, indicating that higher JPE values are associated with reduced postural stability in both age groups (*p* < 0.01).

## 4. Discussion

The main objective of this cross-sectional study was to compare cervical proprioception, functional mobility, and the LOS between individuals above 65 years and those below 65 years old. The secondary aim was to assess the correlation between cervical proprioception, functional mobility, and the LOS. The study found that older individuals had impaired cervical proprioception, reduced functional mobility, and lower LOS compared to the younger group. Additionally, the results showed a statistically significant relationship between the cervical proprioception, functional performance, and LOS tests. These findings provide important insights into the impact of age on cervical proprioception and postural stability and suggest that targeted interventions may be needed to improve functional performance and reduce the fall risk in older individuals.

Previous studies have shown impaired cervical proprioception in older adults [9,32,33,34,35]. Several explanations can account for the reduced proprioception observed in elderly individuals. One of the main reasons is age-related changes in the sensory receptors responsible for proprioception, such as the muscle spindles and joint receptors [10]. These receptors provide information about joint position and movement, and their function deteriorates with age, leading to a decline in proprioceptive acuity [36]. Numerous researchers have investigated the association between the aging process and structural changes in the articular and cutaneous receptors [21,37,38]. In addition, age-related changes in the central nervous system, including the brain and spinal cord, can also contribute to impaired proprioception [26,32,33,34,35,37,39,40,41,42,43,44]. This can result in the slower processing of proprioceptive information, leading to a reduced ability to accurately perceive joint position and movement [10]. Furthermore, degenerative changes in the cervical spine, such as arthritis or disc degeneration, can also contribute to impaired proprioception in the elderly. These changes can affect the function of the sensory receptors responsible for proprioception, leading to reduced acuity and accuracy of joint position sense. Overall, the decline in proprioception in the elderly can be attributed to age-related changes in sensory receptors and central nervous system function, and degenerative changes in the cervical spine. Understanding these factors is important in developing effective interventions to improve proprioception and reduce the risk of falls in older individuals.

Cervical proprioception significantly contributes to balance in older adults. In their study on older adults [45], Quek et al. discovered that either ocular or proprioceptive input was reduced, and the participants’ balance was negatively affected [45]. Reduced proprioception was nearly four times more detrimental to the subjects’ ability to maintain their balance than decreased visual input [45]. Yet, when both proprioception and visual input were reduced, the relative chance of losing balance increased by 5.7 to 7.4 times [43]. Not only does diminished muscular strength affect older adults’ balance, but so does diminished sensory input: the adjusted odds of losing balance dropped by 20% for every Nm/kg increase in the subject’s muscle strength [16].

This study’s results showed that functional mobility and the LOS are compromised in older adults compared to younger ones. As people age, their functional mobility tends to decline, impacting their ability to perform daily activities and increasing their risk of falls [46]. Reduced functional mobility can be attributed to several factors, including age-related declines in muscle strength, flexibility, balance, and co-ordination [47]. One aspect of functional mobility that is particularly important for fall prevention is the individual’s LOS [48], which refer to the boundaries of their safe and stable range of movement while standing or walking [48]. The LOS can be affected by various factors, including age-related declines in proprioception (the ability to sense one’s body position and movements) and vestibular function (the inner ear’s contribution to balance) [48]. Several studies have investigated the relationship between reduced functional mobility and the LOS in elderly individuals [49]. Yacchirema et al. [50] found that older adults with reduced functional mobility demonstrated smaller LOS in both the anterior–posterior and medial–lateral directions compared to younger adults. Phu et al. [48] found that older adults with a history of falls had smaller LOS and longer reaction times than those without a history of falls [48]. The LOS refer to how individuals can control their spatial position and maintain stability in a given posture, per a cited source [16]. According to the results of the present study, older adults exhibit inferior performance compared to younger individuals in both open- and closed-eye trials when posturography is employed to assess postural stability, consistent with prior research [51].

The present study found a strong association between the cervical JPE and measures of the LOS. The study demonstrated that increased proprioceptive errors were linked to longer reaction times, slower maximum excursion, and poorer direction control. These findings are consistent with prior research. Gucmen et al. [52] also reported a significant correlation between cervical active repositioning tests and sway velocities. Reddy et al. [8] found a high correlation between an increased cervical JPE magnitude and postural sway. These results suggest that cervical joint position sense plays a more significant role in regulating dynamic standing balance than static balance, which is critical for fall prevention. Lord et al. [53] found a positive correlation between reaction time and cervical proprioception impairment, with older adults responding more slowly to visual stimuli. Previous research has demonstrated that decreases in the LOS are associated with the aging process and are a significant predictor of multiple falls [8,54]. Fallers have longer reaction times and lesser maximum excursion and direction control parameters than those who have never fallen. These findings highlight the importance of assessing and addressing impairments in cervical proprioception and the LOS to reduce the fall risk in older adults [54].

Cervicogenic dizziness may contribute to balance problems in older adults, as it can interfere with the vestibular system, which is responsible for balance and spatial orientation [55]. One study by Kristjansson et al. [56] investigated the prevalence of cervicogenic dizziness in older adults and its relationship with balance. The study found that cervicogenic dizziness was present in 20% of older adults with balance problems and that those with cervicogenic dizziness had significantly worse balance performance than those without it [56]. The study concluded that cervicogenic dizziness might be important in balance problems among older adults. Another study by Reid et al. (2014) investigated the effects of cervical spine manipulation on balance in older adults with cervicogenic dizziness [57]. The study found cervical spine manipulation improved balance performance in older adults with cervicogenic dizziness [57]. Overall, cervicogenic dizziness may contribute to balance problems in older adults. Further research is needed to better understand the relationship between cervicogenic dizziness and balance and to develop effective interventions for older adults with this condition.

Interventions to improve proprioceptive sensibility, functional mobility, and the LOS in elderly individuals may reduce falls. For example, exercise programs that focus on improving strength, balance, and co-ordination have been shown to improve functional mobility and reduce the risk of falls in older adults [58,59]. Additionally, interventions targeting specific sensory systems, such as visual or vestibular training, may improve the LOS in older adults [60]. In summary, reduced cervical proprioception, functional mobility, and LOS are important factors to consider in evaluating and managing the fall risk in elderly individuals.

Based on the findings of our study, we recommend the following future research avenues: Conducting longitudinal studies can provide valuable insights into the longitudinal changes in cervical proprioception, functional mobility, and the LOS in older adults. Longitudinal designs would allow us to examine the trajectory of these variables over time and identify the factors that contribute to their decline or maintenance. Further investigation is needed to explore the effectiveness of targeted interventions in improving cervical proprioception, functional mobility, and the LOS in older adults. Randomized controlled trials can be conducted to evaluate the impact of exercise programs, sensory training, or other interventions on these parameters and fall risk reduction.

### Limitations of the Study

Our sample discovered that proprioception, functional mobility, and the LOS were compromised in older adults. Even though poor proprioception and mobility are significant intrinsic risk factors for falls, due to our research’s cross-sectional nature, it is impossible to draw any judgments regarding the order in which the events occurred in time. An inherent limitation of our study is that the JPE was represented using an ordinal scale with whole numbers (0, 1, 2, 3, etc.) due to the measurement capabilities of the inclinometer device used. While, theoretically, the JPE could include decimal values, the inclinometer utilized in our study only provided measurements in whole numbers. As a result, the precision and granularity of the JPE measurements may have been limited. This constraint should be acknowledged when interpreting the results, as finer gradations of the JPE may exist that were not captured by our measurement approach. Future studies could explore alternative measurement methods to address this limitation and capture more nuanced variations in joint position errors.

## 5. Conclusions

The study’s results demonstrate that cervical proprioception, functional mobility, and the LOS are negatively impacted in older adults compared to younger individuals. The study found cervical proprioception to be significantly correlated with the LOS and functional mobility in older adults. These findings have important implications for clinicians and rehabilitation therapists working with older adults, highlighting the need to consider these factors when assessing and designing treatment plans.

## Figures and Tables

**Table 1:** Physical and demographic characteristics of study participants.

Variables	Older Group (>65 Years) (*n* = 100)	Younger Group (<65 Years) (*n* = 100)	*p*-Value
Age (years)	69.53 ± 4.32	52.30 ± 4.23	<0.001
Gender (M:F)	59:41	52:48	<0.001
Height (m)	1.68 ± 0.09	1.73 ± 0.05	0.002
Weight (kg)	71.24 ± 5.96	69.58 ± 5.24	0.030
BMI (kg/m^2^)	24.50 ± 3.80	23.38 ± 2.74	0.201

BMI = body mass index.

**Table 2:** Descriptive characteristics of cervical joint position sense, mobility, and LOS tests.

Variables	Older Group (>65 Years) (*n* = 100)	Younger Group (<65 Years) (*n* = 100)	MD	t	95% CI		*p*-Value
					Lower	Upper	
JPE in flexion (°)	4.28 ± 1.29	1.48 ± 1.37	3.00	1.97	2.64	3.36	<0.001
JPE in extension (°)	5.29 ± 1.50	2.16 ± 0.99	3.25	2.13	2.86	3.64	<0.001
JPE in right rotation (°)	4.27 ± 1.38	1.86 ± 0.98	2.43	1.67	2.12	2.74	<0.001
JPE in left rotation (°)	4.02 ± 1.35	1.99 ± 0.83	2.26	1.98	1.97	2.25	<0.001
BBS test score	46.48 ± 4.13	53.11 ± 5.29	−6.53	2.23	−6.87	−6.19	<0.001
TUG test (s)	10.39 ± 1.43	7.86 ± 1.56	1.45	2.34	1.12	1.78	<0.001
Reaction time (s)	1.39 ± 0.29	0.59 ± 0.19	0.79	1.01	0.72	0.87	<0.001
Maximum excursion (%)	5.12 ± 0.41	8.56 ± 0.53	−2.57	−2.64	−2.66	−2.48	<0.001
Direction control (%)	73.58 ± 3.53	88.95 ± 2.73	−14.14	−6.8	−15.17	−13.11	<0.001

JPE = joint position error, BBS = Berg balance scale, TUG = Timed Up and Go, MD = mean difference, and CI = confidence interval.

**Table 3:** Relationship between cervical joint position errors and balance and LOS tests in adults above 65 years (*n* = 100) and below 65 years (*n* = 100).

Groups	Variables		BBS Test Score	TUG Test (s)	Reaction Time (s)	Maximum Excursion (%)	Direction Control (%)
Above 65 years	JPE in flexion (°)	r	−0.68 **	0.53 **	0.71 **	−0.63 **	−0.61 **
	JPE in extension (°)	r	−0.72 **	0.56 **	0.62 **	−0.62 **	−0.62 **
	JPE in right rotation (°)	r	−0.69 **	0.44 **	0.63 **	−0.61 **	−0.59 **
	JPE in left rotation (°)	r	−0.66 **	0.53 **	0.61 **	−0.60 **	−0.53 **
Below 65 years	JPE in flexion (°)	r	−0.76 **	0.63 **	0.74 **	−0.73 **	−0.72 **
	JPE in extension (°)	r	−0.78 **	0.66 **	0.72 **	−0.74 **	−0.73 **
	JPE in right rotation (°)	r	−0.81 **	0.54 **	0.73 **	−0.71 **	−0.71 **
	JPE in left rotation (°)	r	−0.83 **	0.63 **	0.71 **	−0.71 **	−0.74 **

JPE = joint position error, BBS = Berg balance scale, and TUG = Timed Up and Go. ** = Correlation is significant at the 0.01 level (2-tailed).

## Data Availability

All data generated or analyzed during this study are included in this article.
